# A Systematic Review and Meta-Analysis of MIP-1α and MIP-1β Chemokines in Malaria in Relation to Disease Severity

**DOI:** 10.3390/medicina61040676

**Published:** 2025-04-06

**Authors:** Saruda Kuraeiad, Kwuntida Uthaisar Kotepui, Aongart Mahittikorn, Nsoh Godwin Anabire, Frederick Ramirez Masangkay, Polrat Wilairatana, Kinley Wangdi, Manas Kotepui

**Affiliations:** 1 Medical Technology, School of Allied Health Sciences, Walailak University, Tha Sala, Nakhon Si Thammarat 80160, Thailand; saruda.ku@wu.ac.th; 2 Center of Excellence in Tropical Pathobiology, Walailak University, Nakhon Si Thammarat 80160, Thailand; 3 Medical Technology, Faculty of Science, Nakhon Phanom University, Nakhon Phanom 48000, Thailand; 4 Department of Protozoology, Faculty of Tropical Medicine, Mahidol University, Bangkok 10400, Thailand; 5 Department of Biochemistry & Molecular Medicine, School of Medicine, University for Development Studies, Tamale P.O. Box TL 1882, Ghana; 6 West African Centre for Cell Biology of Infectious Pathogens (WACCBIP), Department of Biochemistry, Cell & Molecular Biology, University of Ghana, Accra P.O. Box LG 226, Ghana; 7 Department of Medical Technology, Faculty of Pharmacy, University of Santo Tomas, Manila 1008, Philippines; 8 Department of Clinical Tropical Medicine, Faculty of Tropical Medicine, Mahidol University, Bangkok 10400, Thailand; 9 HEAL Global Research Centre, Health Research Centre, Faculty of Health, University of Canberra, Canberra, ACT 2617, Australia; 10 National Centre for Epidemiology and Population Health, Australian National University, Canberra, ACT 2601, Australia

**Keywords:** systematic review, meta-analysis, macrophage inflammatory protein, MIP-1α, MIP-1β, malaria

## Abstract

*Background and Objectives*: Macrophage inflammatory protein-1α (MIP-1α) and MIP-1β act as signaling molecules that recruit immune cells to sites of infection and inflammation. This study aimed to synthesize evidence on blood levels of MIP-1α and MIP-1β in *Plasmodium*-infected individuals and to determine whether these levels differ between severe and uncomplicated malaria cases. *Materials and Methods*: The study protocol was registered in PROSPERO (CRD42024595818). Comprehensive literature searches were conducted in six databases (EMBASE, MEDLINE, Ovid, Scopus, ProQuest, and PubMed) to identify studies reporting blood levels of MIP-1α and MIP-1β in *Plasmodium* infections and clinical malaria. A narrative synthesis was used to describe variations in MIP-1α and MIP-1β levels between malaria patients and controls and between severe and non-severe malaria cases. Meta-analysis was used to aggregate quantitative data utilizing a random-effects model. *Results*: A total of 1638 records were identified, with 20 studies meeting the inclusion criteria. Most studies reported significantly higher MIP-1α and MIP-1β levels in malaria patients compared to non-malarial controls. The meta-analysis showed a significant elevation in MIP-1α levels in malaria patients (*n* = 352) compared to uninfected individuals (*n* = 274) (*p* = 0.0112, random effects model, standardized mean difference [SMD]: 1.69, 95% confidence interval [CI]: 0.38 to 3.00, *I*^2^: 96.0%, five studies, 626 individuals). The meta-analysis showed no difference in MIP-1α levels between severe malaria cases (*n* = 203) and uncomplicated cases (*n* = 106) (*p* = 0.51, SMD: −0.48, 95% CI: −1.93 to 0.96, *I*^2^: 97.3%, three studies, 309 individuals). *Conclusions*: This study suggests that while MIP-1α and MIP-1β levels are elevated in malaria patients compared to uninfected individuals, these chemokines show a limited ability to differentiate between severe and uncomplicated malaria or predict severe outcomes. Further research is needed to clarify their role in malaria pathogenesis and explore potential clinical applications.

## 1. Introduction

Malaria, a disease caused by *Plasmodium* parasites and spread by female *Anopheles* mosquitoes, continues to pose a significant global health problem, especially in tropical and subtropical parts of the world [1,2]. In 2022, approximately 249 million malaria cases were reported, with the majority occurring within 85 endemic countries [1]. Despite ongoing control and elimination efforts, malaria remains the most significant tropical disease, with its immune response involving both innate and adaptive components [3,4]. When *Plasmodium* infection occurs, dendritic cells, macrophages, and natural killer (NK) cells—key components of the innate immune system—are activated, leading to the secretion of pro-inflammatory cytokines such as interferon-gamma (IFN-γ), tumor necrosis factor-alpha (TNF-α), and interleukin-6 (IL-6), which help control the infection and promote adaptive immune responses [4,5].

Chemokines are essential in the immune response to infections, functioning as signaling molecules that recruit immune cells to infection sites and coordinate immune responses [6,7,8]. Several chemokines have been associated with *Plasmodium* infections, including CC chemokine ligand (CCL)2, CCL3, CCL4, CCL5, C-X-C motif chemokine ligand (CXCL)8, CXCL9, CXCL10, CXCL13, and CXCL16 [9,10]. Among these chemokines, macrophage inflammatory protein-1 alpha (MIP-1α or CCL3) and macrophage inflammatory protein-1 beta (MIP-1β or CCL4) belong to the MIP-1 CC chemokine subfamily [11,12]. These proteins act through G-protein-coupled cell surface receptors and contribute to inflammation and cell migration [11]. A prior study indicated that MIP-1α could be a diagnostic marker for identifying various inflammatory diseases and conditions such as rheumatoid arthritis, sarcoidosis, periodontitis, multiple myeloma, respiratory disease, and cardiovascular disease [13].

Regarding the functions of MIP-1α and MIP-1β in *Plasmodium* infections, previous *in vitro* studies have shown that hemozoin crystals strongly stimulate the release of several fever-inducing cytokines, including MIP-1α and MIP-1β [14,15]. Additional evidence on the *in vitro* stimulation of parasitized human red blood cells with hemozoin crystals demonstrated an elevation in levels of various cytokines and chemokines, including MIP-1α and MIP-1β [14]. Previous studies using human subjects have highlighted the roles of MIP-1α and MIP-1β in individuals infected with *Plasmodium* and those with clinical malaria [16,17]. Particularly, an elevation in blood levels of MIP-1α and MIP-1β among individuals with *Plasmodium* infection increases the risk of severe complications, such as severe malarial anemia [17] and cerebral malaria [18,19]. While MIP-1α and MIP-1β have been reported to be associated with *Plasmodium* infection and severe complications, some studies suggest no consistent association between these chemokines and different disease outcomes [18,19]. These discrepancies may be attributed to variations in measurement methods, differences in study populations, or varying definitions of disease severity. Therefore, this study aimed to collate and analyze evidence on blood levels of MIP-1α and MIP-1β in *Plasmodium*-infected individuals and to determine whether these levels differ between severe and uncomplicated malaria cases.

## 2. Methods

### 2.1. Protocol of Systematic Review

The protocol of this study was registered in PROSPERO (registration number CRD42024595818). This study followed the Preferred Reporting Items for Systematic Reviews and Meta-Analyses (PRISMA) guidelines [20].

### 2.2. Definitions

A severe case of *Plasmodium falciparum* infection is characterized by the presence of asexual parasitemia, accompanied by one or more complications, such as hypoglycemia, hyperparasitemia, acidosis, prostration, impaired consciousness, renal failure, pulmonary edema, significant bleeding, shock, multiple convulsions, or severe malarial anemia. Severe malaria caused by *P. vivax* or *P. knowlesi* is defined similarly to *P. falciparum* malaria; however, *P. vivax* lacks a specific threshold for parasite density, while *P. knowlesi* includes one [21]. Non-severe malaria is classified as cases with parasitemia but lacking the complications seen in severe malaria.

### 2.3. The Research Question for This Systematic Review

The research question for this study was structured using the Population, Exposure, Comparator, Outcome (PECO) framework [22]. P represents individuals in endemic malaria areas; E represents an infection with *Plasmodium*, including severe cases; C represents individuals that are either uninfected or with non-severe malaria cases (uncomplicated malaria or asymptomatic infection); O represents blood levels of MIP-1α and MIP-1β. The primary objective was to investigate differences in blood MIP-1α/MIP-1β levels between individuals infected with *Plasmodium* and those without the infection. A secondary objective focused on changes in blood levels of MIP-1α and MIP-1β in individuals with severe malaria versus those with non-severe cases.

### 2.4. Database Searches

A comprehensive literature search was performed in six databases (EMBASE, MEDLINE, Ovid, Scopus, ProQuest, and PubMed), to identify articles that reported blood levels of MIP-1α and MIP-1β in cases of *Plasmodium* infection and clinical malaria. A search in these databases was employed using the following groups of key terms; “Macrophage Inflammatory Proteins”, “chemokine (C-C motif) ligand 3”, “C-C motif chemokine ligand 4”, and “malaria”. The keyword searches were adjusted slightly per database according to the guidelines of each (as detailed in Tables S1–S3). The literature was searched until 29 September 2024, with no publication date or language restrictions. Additionally, Google Scholar was searched to ensure coverage of non-indexed studies. The reference list was reviewed to minimize missing relevant articles.

### 2.5. Eligibility Criteria

The inclusion criteria focused on human studies that assessed and reported data on blood levels of MIP-1α and MIP-1β in cases of *Plasmodium* infection and clinical malaria. Studies with a cross-sectional, cohort, or case–control design were considered. Exclusion criteria included *in vitro* studies, animal models, and non-original articles such as reviews, meta-analyses, case reports, case series, commentaries, or letters.

### 2.6. Study Selection and Data Extraction

Once the articles were retrieved from the databases, duplicates were removed first by automated processes, followed by manual review using Endnote (version 20.0, Clarivate, Philadelphia, PA, USA). Titles and abstracts were independently screened for relevance, and full texts of potentially eligible articles were assessed based on the established eligibility (inclusion and exclusion) criteria. Any study failing to meet the requirements was excluded, with reasons provided. Data were extracted from eligible studies, focusing on characteristics such as publication year, geographic region (country), study design (cross-sectional, cohort, or case–control design), participant demographics (mean age, age range), malaria diagnostic methods (microscopic method, rapid diagnostic test (RDT), or molecular method), MIP-1α/MIP-1β assessment methods (enzyme-linked immunosorbent assay [ELISA], bead-based assay, or other methods), and the type of blood specimen (plasma or serum) utilized for MIP-1α/MIP-1β assessment. Two authors (SK and MK) conducted the selection and extraction independently, resolving disagreements through discussion.

### 2.7. Risk of Bias Assessment

The risk of bias was assessed using the Joanna Briggs Institute (JBI) critical appraisal tools [23]. For cross-sectional studies, the appraisal concentrated on the transparency of the inclusion guidelines, consistency of exposure and outcome measures, control of confounders, and appropriateness of statistical analyses. In case–control studies, the evaluation included case and control selection, confounder identification, accuracy in exposure measurement, and statistical adequacy. For cohort studies, aspects such as participant selection, confounder management, accuracy of outcome measurements, and consistency of follow-up were considered. Two researchers (SK and MK) evaluated the bias risk independently, with conflicts settled by a third researcher (AM).

### 2.8. Data Synthesis

The data synthesis approach was based on previous research methods [24,25]. A narrative synthesis was employed to describe changes in blood levels of MIP-1α and MIP-1β between individuals with malaria and those without, as well as between severe and non-severe cases. A meta-analysis was employed to aggregate quantitative data on blood levels of MIP-1α and MIP-1β, utilizing a random effects model [25]. A fixed-effects model was used in parallel as part of a sensitivity analysis to validate the consistency of the findings. Heterogeneity was presented by the *I*^2^ statistics, with values above 50% suggesting a considerable heterogeneity [26]. A subgroup analysis was conducted to assess whether study design, geographic region, participant age, *Plasmodium* species, diagnostic methods, and the type of blood specimen for MIP-1α/MIP-1β influenced the heterogeneity. The impact of individual studies was evaluated using influence analysis [27]. If at least 10 articles were included in the meta-analysis, a funnel plot and Egger’s test were conducted to assess the publication bias of the result [28]. The meta-analysis was conducted using RStudio (Version: 2024.04.2 + 764) [29], with significance set at *p* < 0.05.

## 3. Results

### 3.1. Search Results

**Figure 1:**
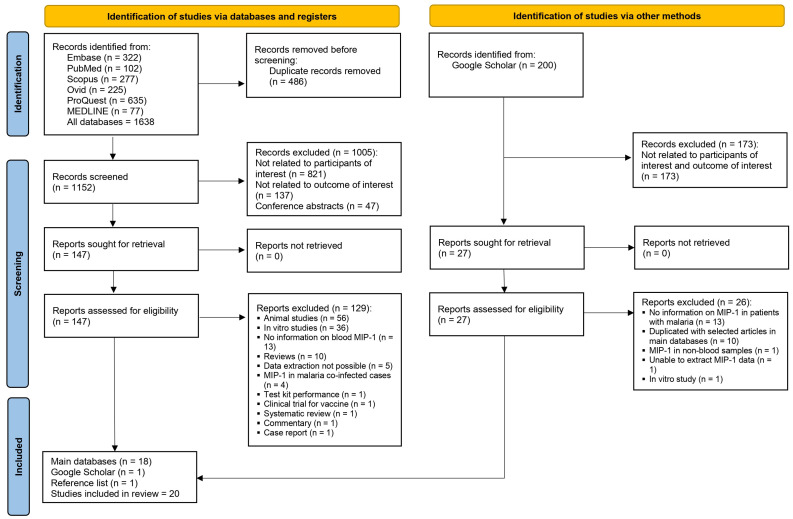
Study flow diagram.

A total of 1638 records were retrieved from Embase (*n* = 322), PubMed (*n* = 102), Scopus (*n* = 277), Ovid (*n* = 225), ProQuest (*n* = 635), and MEDLINE (*n* = 77). After removing 486 duplicate records, 1152 unique records remained for screening. The initial screening excluded 1005 records for reasons such as lack of relevance to the participants or outcomes of interest (*n* = 821), unrelated outcomes (*n* = 137), and being conference abstracts (*n* = 47). After screening, 147 articles were searched for full-text and eligibility assessment. Of the 147 reports assessed, 129 reports were excluded after eligibility checks for reasons such as an animal study focus (*n* = 56), *in vitro* studies (*n* = 36), lack of blood MIP-1 information (*n* = 13), being reviews or systematic reviews (*n* = 10), and cases where data extraction was not possible (*n* = 5), and other reasons (*n* = 9). Additionally, 200 records were identified from Google Scholar, of which 173 were excluded as they were not focused on the participants or outcomes. A further 26 studies that lacked information on MIP-1 in malaria patients (*n* = 13) or where there was a duplication with selected articles (*n* = 10), use of non-blood samples (*n* = 1), inability to extract MIP-1 data (*n* = 1), or focus on *in vitro* studies (*n* = 1) were excluded. Finally, 20 studies, sourced from the main databases (*n* = 18) [17,18,19,30,31,32,33,34,35,36,37,38,39,40,41,42,43,44], Google Scholar (*n* = 1) [45], and reference lists (*n* = 1) [46], were included in the review (Figure 1).

### 3.2. Characteristics of Included Studies

The included studies were published between 1995 and 2023 (Table 1). Regarding the study design, the included studies comprised cohort studies (eight studies, 40%) [17,18,35,38,39,43,44,45], case–control studies (eight studies, 40%) [19,31,36,37,40,41,42,46], and cross-sectional studies (four studies, 20%) [30,32,33,34]. The studies were primarily conducted in Africa (fourteen studies, 70%) [17,18,30,31,33,34,36,38,41,42,43,44,45,46], followed by Asia (four studies, 20%) [19,35,37,40], South America (one study, 5%) [32], and multi-continents (one study, 5%) [39]. Participants included children (nine studies, 45%) [17,18,31,33,41,42,43,45,46], adults (four studies, 20%) [34,35,36,37,38], and pregnant women (five studies, 25%) [30,32,36,39,44], and two studies covered all age groups (10%) [19,40].

The included studies focused on participants with different severities of *Plasmodium* spp. infection: both severe and uncomplicated malaria (eleven studies, 55%) [17,18,19,33,34,37,38,40,41,42,45], severe malaria only (three studies, 15%) [31,35,43], uncomplicated malaria only (one study, 5%) [36], symptomatic malaria (unspecified severe or uncomplicated, one study, 5%) [46], and an unspecified clinical status (four studies, 20%) [30,32,39,44]. Most studies investigated *P. falciparum* only (sixteen studies, 80%) [17,18,19,30,31,33,35,36,38,40,41,42,43,44,45,46], with a few examining *P. falciparum* and non-*P. falciparum* infections (three studies, 15%) [32,34,37] or *P. vivax* only (one study, 5%) [39]. For malaria detection, microscopy was the primary method used (thirteen studies, 65%) [17,18,19,30,31,33,35,36,38,41,42,44,45], with some studies combining microscopy with PCR and/or RDT (seven studies, 35%) [32,34,37,39,40,43,46]. For MIP-1 detection, methods included bead-based assays (thirteen studies, 65%) [18,19,30,32,34,38,39,40,41,42,43,45,46] and ELISA (seven studies, 35%) [17,31,33,35,36,37,44]. Most studies collected plasma samples (fifteen studies, 75%) [17,19,30,32,33,34,36,39,40,41,42,43,44,45,46], while the remaining studies used serum samples (five studies, 25%) [19,31,35,37,38] for MIP-1 detection.

### 3.3. Risk of Bias

Almost all the cross-sectional studies [30,32,33,34] met the criteria for clearly defining inclusion in the study, except for one study [33] that was unclear about strategies to address confounding factors. Most case–control studies [19,31,36,37,40,41,42,46] met the criteria for group comparability. However, four studies [36,37,46] did not identify confounding factors or state strategies to address them.

All prospective/cohort studies [17,18,35,38,39,43,44,45] demonstrated a similarity in groups recruited from the same population, consistent exposure measurement for both exposed and unexposed groups, valid and reliable exposure measurement, and an appropriate statistical analysis. Most studies identified confounding factors, but some studies did not [17,35], and strategies to address confounding factors were only reported by some studies [18,38,39,43,45]. Several studies [17,18,45] did not report follow-up times, while others [17,18,35,38,45] were unclear about the completeness of the follow-up or reasons for loss to follow-up. Most studies did not provide strategies to address incomplete follow-up, with only one study reporting such strategies [39].

### 3.4. MIP-1α in Participants with Plasmodium Infections

Fifteen studies reported MIP-1α levels in malaria patients and uninfected controls [17,18,19,30,31,32,33,34,35,36,38,40,41,44,46]. In non-pregnant individuals, six studies demonstrated significantly higher MIP-1α levels among malaria patients than non-malarial individuals [17,18,35,38,40,46]. One study reported a notable elevation in MIP-1α in malaria cases compared to endemic controls [40]. One study found that MIP-1α levels at admission were higher in malaria patients than in healthy controls; however, the significance of the difference between groups was unclear [35]. Additionally, MIP-1α levels were significantly elevated in malaria patients (including non-cerebral and/or cerebral malaria) compared to control individuals [38]. One study observed that the mean MIP-1α levels were higher in malaria patients than in febrile controls, although no significant difference was reported [46]. Cerebral malaria patients exhibited a significantly higher MIP-1α level than community controls [18]. Furthermore, MIP-1α levels were significantly elevated in malaria cases compared to healthy controls, whether in severe or uncomplicated cases [17]. In another study, MIP-1α was significantly elevated in severe malaria cases compared to non-malarial febrile individuals, but no significant differences were observed between uncomplicated malaria and febrile controls [41]. In contrast, four studies reported no notable alteration in MIP-1α levels between malaria patients and non-malarial individuals [19,31,33,34]. Specifically, no differences were observed in MIP-1α levels between malaria cases (including cerebral malaria and severe malarial anemia) and non-malarial controls [31]. Similarly, no differences were reported between uncomplicated or cerebral malaria and healthy controls [33,34]. Another study found no differences in MIP-1α levels between malaria patients (including mild malaria patients and cerebral malaria survivors/non-survivors) and healthy controls [19].

Among pregnant women, significantly higher MIP-1α levels were observed in pregnant women with *Plasmodium* infections than those without [30,32]. However, no significant difference in MIP-1α levels was found between HIV-negative/placental malaria (PM)-negative and HIV-negative/PM-positive individuals [36]. Similarly, no differences in MIP-1α/MIP-1β levels were found between pregnant women with *Plasmodium* infections and those without the infection [44].

### 3.5. MIP-1β in Plasmodium Infections

Fourteen studies reported MIP-1β levels in malaria cases compared to non-malarial individuals [17,18,19,30,31,33,34,36,38,39,40,41,44,46]. Among non-pregnant individuals, three studies demonstrated significantly higher MIP-1β levels in malaria cases than in non-malarial individuals [17,31,41]. Specifically, MIP-1β levels were significantly elevated in malaria in both uncomplicated and severe patients compared to healthy individuals [17]. One study showed significantly elevated MIP-1β levels in patients with severe malaria compared to non-malarial febrile individuals but found no notable alteration between patients with uncomplicated malaria and febrile individuals [41]. Another study observed significantly higher MIP-1β levels in malaria patients (including severe malarial anemia and cerebral malaria) in comparison to non-malaria cases [31]. Additionally, one study indicated that MIP-1β levels were not statistically increased in malaria cases compared to febrile individuals without malaria [46]. In contrast, two studies found no notable alteration in MIP-1β levels between patients with malaria and non-malarial cases [33,40]. Additionally, no significant differences were observed in MIP-1β levels between malaria patients (including those with cerebral malaria, severe malarial anemia, and uncomplicated malaria) and healthy controls [33]. Similarly, no differences were observed between malaria patients and endemic controls [40]. One study revealed significantly higher MIP-1β levels in malaria patients (specifically mild malaria or cerebral malaria survivors) in comparison to healthy individuals. Still, no notable alteration was found between cerebral malaria non-survivors and healthy controls [19].

Among pregnant women, significantly higher MIP-1β levels were demonstrated in malaria cases in comparison to non-malarial individuals [30]. MIP-1β levels were also significantly higher in individuals with *P. vivax* infections than those without the infection at the time of recruitment [39]. On the contrary, MIP-1β was observed to be altered in HIV-negative PM-negative compared to HIV-negative PM-positive pregnant women [36]. Similarly, no notable alteration in MIP-1β levels was found between pregnant women with malaria and those without the infection [44].

### 3.6. MIP-1α/MIP-1β Between Participants with Severe and Uncomplicated Malaria

Nine studies reported MIP-1α levels in patients with severe and uncomplicated malaria [17,18,19,33,34,40,41,42,45]. Most found no significant difference in MIP-1α levels between severe and uncomplicated or mild malaria cases [18,19,34,40,41,42,45]. One study reported a non-significant increase MIP-1α levels in severe malaria cases compared to uncomplicated cases [17]. Another study found significantly lower MIP-1α levels in cerebral malaria compared to uncomplicated cases, with no alteration in severe malarial anemia compared to uncomplicated malaria cases [33].

Eight studies enrolling severe and uncomplicated malaria cases reported MIP-1β levels [17,18,19,33,34,40,41,42]. Most of these studies observed no notable alteration in MIP-1β levels between severe malaria and uncomplicated or mild malaria patients [18,19,33,40,41,42]. One study observed significantly higher MIP-1β levels in severe malaria cases than in uncomplicated malaria cases [34]. Another study found a non-significant increase in MIP-1β in severe malaria cases compared to mild malaria [17].

### 3.7. MIP-1α/MIP-1β Between Different Severe Malarial Complications, Fatality, and Plasmodium Species

For different severe malarial complications, the reviewed studies showed no significant differences in MIP-1α or MIP-1β levels between malaria cases with severe anemia and cerebral malaria [31,33,45]. One study observed no difference in MIP-1α or MIP-1β levels between severe and non-severe malarial anemia cases [42]. Another study reported no differences in MIP-1α or MIP-1β levels between malaria cases with multiorgan dysfunction and those with severe non-cerebral malaria [40].

Regarding fatality status, one study found no differences in MIP-1α or MIP-1β levels among cerebral malaria survivors in comparison to non-survivors [19]. Another study similarly observed no difference in MIP-1α or MIP-1β levels between cerebral malaria patients who died and those who survived [43]. However, a separate study found that both MIP-1α and MIP-1β levels were significantly lower in survivors compared to cerebral malaria patients who died [38].

For different *Plasmodium* species, one study reported that *P. knowlesi*-infected individuals had significantly lower MIP-1β levels compared to *P. falciparum*-infected individuals, but MIP-1β levels were comparable among patients with *P. knowlesi* and *P. vivax* malaria [37]. Additionally, there was no significant difference in MIP-1β levels between *P. vivax* and *P. falciparum* groups [37].

### 3.8. Meta-Analysis of MIP-1α/MIP-1β in Malaria Cases and Uninfected Individuals

**Figure 2:**
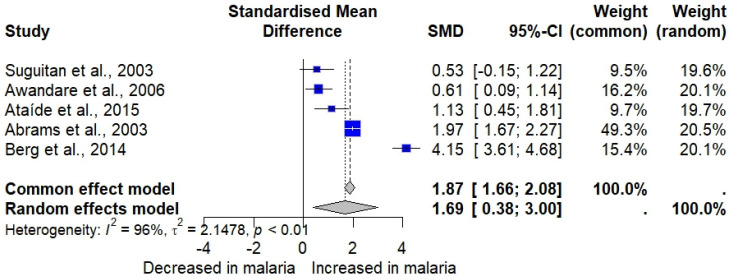
The forest plot shows the standardized mean difference (SMD) in MIP-1α levels between malaria patients and uninfected individuals across five studies [30,32,33,34,44]. Each study is represented by a blue square, with the size reflecting its weight in the meta-analysis. Horizontal lines denote each study’s 95% confidence intervals (CIs). The pooled effect sizes are presented under the common (random) and random effects models, with the overall SMD indicated by a diamond. Black vertical line is no effect line. Black-dashed lines are the overall SMD by common (fixed-) and random-effects models. High heterogeneity was observed among the studies (*I²* = 96%, *p* < 0.01).

Five studies assessing MIP-1α in malaria patients and uninfected individuals [30,32,33,34,44] were included in the meta-analysis. The results showed a significant elevation in MIP-1α in malaria patients (*n* = 352) compared to uninfected individuals (*n* = 274) (*p* = 0.0112, random effects model, standardized mean difference [SMD]: 1.6866, 95% confidence interval [CI]: 0.3777 to 2.9955, *I*^2^: 96.0%, number of studies: five, 626 individuals, Figure 2). Subgroup analysis revealed that participant variations and differences in diagnostic methods significantly altered the pooled results (*p* < 0.05, Table S4).

**Figure 3:**
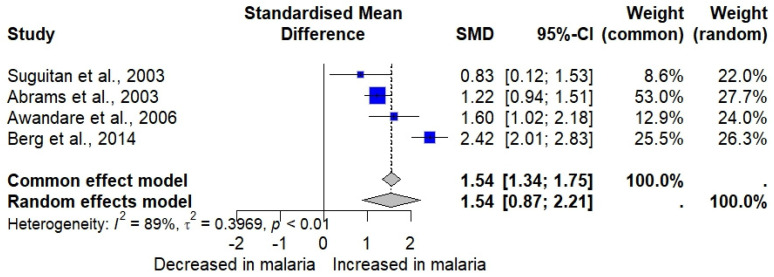
The forest plot shows the standardized mean difference (SMD) in MIP-1β levels between malaria patients and uninfected individuals across four studies [30,33,34,44]. Each study is represented by a blue square, with the size reflecting its weight in the meta-analysis. Horizontal lines denote each study’s 95% confidence intervals (CIs). The pooled effect sizes are presented under the common (random) and random effects models, with the overall SMD indicated by a diamond. Black vertical line is no effect line. Black-dashed lines are the overall SMD by common (fixed-) and random-effects models. High heterogeneity was observed among the studies (*I²* = 89%, *p* < 0.01).

Four studies assessing MIP-1β in malaria patients and uninfected individuals [30,33,34,44] were included in the meta-analysis. The results showed a significant elevation in MIP-1β in malaria cases (*n* = 304) compared to uninfected individuals (*n* = 241) (*p* < 0.0001, random effects model, SMD: 1.5413, 95% CI: 0.8744 to 2.2082, *I*^2^: 88.8%, number of studies: four, 545 individuals, Figure 3). Subgroup analysis revealed that differences in participant characteristics, *Plasmodium* species, malaria diagnostic methods, and MIP-1β assays significantly altered the pooled results (*p* < 0.05, Table S4).

### 3.9. Meta-Analysis of MIP-1α/MIP-1β in Severe and Uncomplicated Malaria Cases

**Figure 4:**
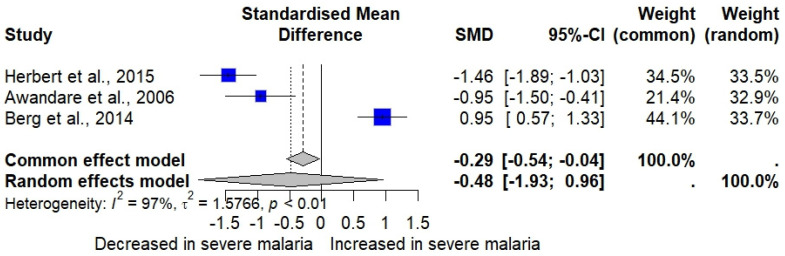
The forest plot shows the standardized mean difference (SMD) in MIP-1α levels between patients with severe and uncomplicated malaria across three studies [33,34,40]. Each study is represented by a blue square, with the size reflecting its weight in the meta-analysis. Horizontal lines denote each study’s 95% confidence intervals (CIs). The pooled effect sizes are presented under the common (random) and random effects models, with the overall SMD indicated by a diamond. Black vertical line is no effect line. Black-dashed lines are the overall SMD by common (fixed-) and random-effects models. High heterogeneity was observed among the studies (*I*² = 97%, *p* < 0.01).

The meta-analysis of three studies reporting MIP-1α levels in severe and uncomplicated malaria cases [33,34,40] revealed no significant difference in MIP-1α levels between severe malaria cases (*n* = 203) and uncomplicated malaria cases (*n* = 106) (*p* = 0.5135, random effects model, SMD: −0.4817, 95% CI: −1.9268 to 0.9634, *I*^2^: 97.3%, number of studies: three, 309 individuals, Figure 4). A subgroup analysis was not conducted because of the limited number of studies.

**Figure 5:**
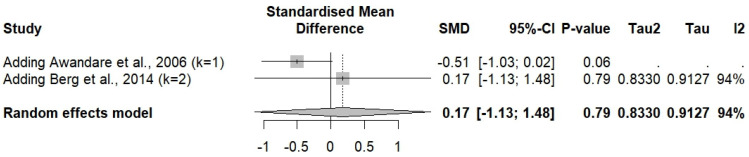
The forest plot shows the standardized mean difference (SMD) in MIP-1β levels between patients with severe and uncomplicated malaria across two studies [33,34]. Each study is represented by a gray square, with the size reflecting its weight in the meta-analysis. Horizontal lines denote each study’s 95% confidence intervals (CIs). The pooled effect sizes are presented under the common (random) and random effects models, with the overall SMD indicated by a diamond. Black vertical line is no effect line. Black-dashed lines are the overall SMD by random-effects model. High heterogeneity was observed among the studies (*I²* = 94%).

The meta-analysis of two studies reporting MIP-1β levels in severe and uncomplicated malaria cases [33,34] revealed no significant difference in MIP-1β levels between severe malaria cases (*n* = 120) and uncomplicated malaria cases (*n* = 69) (*p* = 0.7943, random effects model, SMD: 0.1736, 95% CI: −1.1316 to 1.4788, *I*^2^: 93.9%, number of studies: two, 189 individuals, Figure 5). A subgroup analysis was not conducted because of the limited number of studies.

### 3.10. Sensitivity Analysis

The meta-analysis using the fixed-effect model demonstrated similar findings to the random effects model, in which a significant elevation of MIP-1α in malaria cases in comparison to uninfected participants was observed (*p* < 0.001, SMD: 1.8688, 95% CI: 1.6576 to 2.0801, Figure 2). Influence analysis revealed that removing one study [30] affected the pooled effect estimate after rerunning the meta-analysis (*p*: 0.0615).

The meta-analysis using the fixed-effects model demonstrated similar findings to the random effects model, showing a significant elevation in MIP-1α malaria cases compared to uninfected participants (*p* < 0.001, SMD: 1.5424, 95% CI: 1.3359 to 1.7488, Figure 3). The influence analysis suggested that no single study significantly altered the pooled results after rerunning the meta-analysis (*p* < 0.05 in all rerun analyses).

The meta-analysis using the fixed-effects model showed findings contrary to the random effects model, indicating a significant elevation in MIP-1α in severe malaria cases compared to uncomplicated malaria cases (*p* = 0.0249, SMD: −0.2892, 95% CI: −0.5419 to −0.0365, Figure 4). The influence analysis suggested that omitting Berg et al., 2014 [34] significantly altered the pooled results after rerunning the meta-analysis (*p* < 0.001).

### 3.11. Publication Bias

Publication bias was not assessed because each meta-analysis included fewer than 10 studies.

## 4. Discussion

This systematic review and meta-analysis evaluated the levels of MIP-1α and MIP-1β in participants with *Plasmodium* infections, including comparisons between malaria and non-malaria cases, severe and uncomplicated malaria cases, and different severe malaria complications, fatality statuses, and *Plasmodium* species. Significantly elevated levels of MIP-1α and MIP-1β were observed in malaria patients compared to uninfected controls, as supported by meta-analysis findings (*p* = 0.0112 for MIP-1α, *p* < 0.0001 for MIP-1β). These results suggest that MIP-1α and MIP-1β amplify the inflammatory response in *Plasmodium* infections by acting as monocyte chemoattractants and contributing to infection-induced inflammation [11,47]. They activate monocytes/macrophages to produce pro-inflammatory cytokines such as TNF-α, IL-6, and IL-1α [48]. These cytokines, in turn, induce acute-phase proteins, promote the further production of cytokines, and recruit immune cells to sites of inflammation [49,50].

The high *I*^2^ values (95% for MIP-1α, 88.8% for MIP-1β) indicate substantial variability, likely due to study characteristics and the limited number of studies included in the meta-analysis. For MIP-1α, subgroup analyses (with few studies) showed that participant types, diagnostic methods, and assay types significantly influenced the pooled outcomes. For MIP-1β, subgroup analyses identified participant types, *Plasmodium* species, diagnostic methods, and assay types as significant sources of heterogeneity among the studies. Among participant types, adults appear to have a higher SMD of MIP-1α and MIP-1β compared to children and pregnant women, with an overall trend of increased MIP-1α and MIP-1β levels observed across all groups. The variations in the types of participants enrolled in each study may explain the variability in MIP-1α and MIP-1β levels. Adults typically have a higher immune response to malaria due to repeated infections compared to children whose immune systems are not yet fully developed [51,52]. Meanwhile, pregnant women may be more susceptible to *Plasmodium* infections, with inflammatory responses in the placenta frequently observed, which may alter the cytokine balance in the fetus [53,54]. In pregnant women, activated macrophages in the placenta may produce more MIP-1β, and MCP-1 may, in turn, further attract monocytes to the placenta [30]. Increased MIP-1α and MIP-1β levels have also been associated with a higher parasite density, as previous studies have shown that malaria pigments can induce MIP-1α and MIP-1β production [14,36]. Together, these results suggest that pregnant women may have more activated immune cells producing MIP-1β than other groups. However, the limited number of studies in the subgroup analysis may constrain this interpretation.

The subgroup analysis suggests that different *Plasmodium* species may influence the outcomes. For MIP-1α, *P. falciparum* infections exhibited a significantly higher SMD than non-*P. falciparum* infections, which showed no significant changes in MIP-1α levels. In contrast, for MIP-1β, both *P. falciparum* and non-*P. falciparum* infections showed a significant increase in SMD. These findings suggest that MIP-1β may respond to infections with multiple *Plasmodium* species, whereas MIP-1α appears more specific to *P. falciparum* infections. The reviewed study reported lower MIP-1β levels in *P. knowlesi*-infected patients compared to *P. falciparum*, while no differences were observed between *P. vivax* and *P. falciparum*. Distinct *Plasmodium* species may contribute to subtle differences in macrophage subset activation, potentially affecting disease severity [37]. In the context of different *Plasmodium* species infections, variations in MIP-1α and MIP-1β can be linked to the differing complication severity caused by *Plasmodium*, such as renal impairment in *P. knowlesi* infections and severe anemia in *P. falciparum* [37]. This difference may be attributed to the distinct immune responses elicited by *P. falciparum*, which induces stronger inflammatory cytokine and chemokine activation due to its ability to sequester itself in the microvasculature and cause severe pathology [55]. In contrast, non-*P. falciparum* species typically induce a less pronounced inflammatory response, which may explain the lack of significant MIP-1α elevation in these infections. The broader increase in MIP-1β across species suggests a more general role in the immune response to *Plasmodium* infections, potentially contributing to leukocyte recruitment and pathogen clearance.

For alterations in MIP-1α and MIP-1β in severe versus uncomplicated malaria cases, the findings showed no significant differences in MIP-1α or MIP-1β levels between severe and uncomplicated malaria cases based on the random-effects model meta-analyses. However, sensitivity analyses using fixed-effects models suggested significant differences for MIP-1α. This discrepancy suggests potential biases due to a few studies included in the meta-analysis, small sample sizes, and study heterogeneity. The meta-analysis results suggest that while the chemokine (MIP-1α and MIP-1β) levels are elevated in malaria, they may not clearly differentiate disease severity. The role of MIP-1α and MIP-1β in severe malaria remains unclear. However, few studies have reported increased MIP-1β levels in the plasma and cerebrospinal fluid (CSF) of children with cerebral malaria [19,31].

Based on the current findings that show no differences in MIP-1α or MIP-1β levels between severe complications, MIP-1α and MIP-1β levels may not be specific to severe malaria or may not be specific markers of severe malaria complications, possibly due to differentially enhanced humoral and cellular immune responses [56]. The limited data suggest no association with malaria fatality for MIP-1α and MIP-1β alterations in fatal versus non-fatal cases. While one study reported significantly lower levels of MIP-1α and MIP-1β in cerebral malaria survivors compared to non-survivors [38], others found no differences [19,43]. These findings suggest that MIP-1α and MIP-1β may not directly affect malaria-related fatality. However, other chemokines and cytokines, such as TNF-α, IL-6, and IFN-γ, may significantly drive severe disease outcomes and mortality.

Beyond *Plasmodium* infections, MIP-1α and MIP-1β have been implicated in other protozoan parasitic diseases. For instance, in *Trypanosoma cruzi*, the causative agent of Chagas disease. Increased MIP-1β levels have been associated with heightened inflammation and disease progression, particularly in the chronic cardiac form of the disease [57]. In *Toxoplasma gondii* infections, MIP-1α plays a key role in recruiting immune cells to sites of infection, with elevated levels linked to stronger resistance in murine models [58]. Furthermore, research suggested that MIP-1α stimulates strong antigen-specific serum immunoglobulin responses. At the same time, MIP-1β promotes higher mucosal IgA and IgE antibody responses, suggesting that these chemokines differentially mediate mucosal and systemic adaptive immunity [57], which may be relevant in various parasitic infections.

In other diseases, MIP-1α and MIP-1β were elevated in sepsis cases and are associated with poor clinical outcomes [9,10,59]. Elevated levels of these cytokines were also observed in HIV-infected patients compared to HIV-uninfected individuals [60]. A previous study found that MIP-1α and MIP-1β are major HIV-suppressive factors produced by CD8+ T cells, playing a role in controlling HIV infection *in vivo* [61]. MIP-1α has also been implicated in post-COVID-19 patients, where it predicts post-COVID-19 symptomatology [62]. Furthermore, MIP-1α and MIP-1β have been associated with several inflammatory diseases, including periodontitis, chronic rhinosinusitis, rheumatoid arthritis, Sjögren’s syndrome, multiple myeloma, and nasopharyngeal carcinoma [63,64].

This study’s strengths include a comprehensive review of two chemokine levels across diverse malaria populations and robust meta-analysis methods. However, high heterogeneity and limited data on specific subgroups limit the generalizability of the findings. The leave-one-out sensitivity analyses revealed that excluding certain studies significantly altered the overall effect size, suggesting that individual studies contributed substantially to the observed heterogeneity. This variability may be due to differences in study design, population characteristics, or measurement techniques. Additionally, subgroup analyses of MIP-1α and MIP-1β levels between severe and uncomplicated malaria were not conducted due to limited studies. Publication bias was also not assessed because the meta-analysis included fewer than 10 studies. Furthermore, as most of the included studies were conducted in Africa (70%), the findings may be less generalizable to other regions, highlighting the need for more research in diverse geographical settings. Future research should focus on larger sample sizes and explore the role of MIP-1α and MIP-1β concerning other immune markers to improve the understanding of their association with severe malaria’s pathophysiology and inform prognostic tool development.

## 5. Conclusions

This systematic review and meta-analysis suggest an elevation in levels of MIP-1α and MIP-1β in malaria patients compared to uninfected individuals. However, these chemokines show a limited ability to differentiate between severe and uncomplicated malaria, various severe complications, or fatal and non-fatal outcomes, partly due to the limited number of studies investigating these markers. While MIP-1α and MIP-1β can be viewed as potential diagnostic markers for malaria, their prognostic utility remains unclear. Further research is needed to elucidate their role in malaria pathogenesis and explore their clinical applications. 

## Figures and Tables

**Table 1:** Results of individual studies reporting blood levels of MIP-1α and MIP-1β in participants with *Plasmodium* infections.

No.	Authors	Study Location (Conduction Year)	Participants (Number)	*Plasmodium* Species	Type of Malaria (i.e., Uncomplicated, Severe)	Method for Malaria Detection	Blood Samples for MIP-1 Detection	Method for MIP-1 Detection	Qualitative Blood Levels of MIP-1α and MIP-1β
1	Abrams et al., 2003 [30]	Malawi (1998–2000)	Pregnant women (537)	*P. falciparum*	Not specified	Microscopic method	Plasma	ELISA (Quantikine sandwich ELISA kits, R&D Systems, Abingdon, Oxon, UK.)	MIP-1α: Significantly higher MIP-1α levels were observed in malaria-infected individuals than uninfected individuals. MIP-1β: Significantly higher MIP-1β levels were observed in malaria-infected individuals than uninfected individuals.
2	Armah et al., 2007 [31]	Ghana (2005)	Children: Cerebral malaria (9), severe malarial anemia (5), non-malaria deaths/non-malarial controls (5)	*P. falciparum*	Severe malaria	Microscopic method	Serum	Commercially available multiplex colorimetric bead-based cytokine immunoassay coupled with the Luminex™ system (Austin, TX, USA) and human-specific bead sets (BioRad, San Diego, CA, USA)	Blood samples: MIP-1α: No difference in MIP-1α levels between cerebral malaria, severe malarial anemia, and non-malarial controls. MIP-1β: No difference in MIP-1β levels between cerebral malaria, severe malarial anemia, and non-malarial controls. CSF samples: MIP-1α: No difference in MIP-1α levels between cerebral malaria, severe malarial anemia, and non-malarial controls. MIP-1β: Significantly higher MIP-1β levels were observed in severe malarial anemia compared to non-malaria cases. Significantly higher MIP-1β levels were observed in cerebral malaria compared to non-malaria cases. No difference in MIP-1β levels between cerebral malaria and severe malarial anemia cases.
3	Ataíde et al., 2015 [32]	Brazil (2012–2013)	Pregnant women (137): Infected (45), uninfected (92)	*P. falciparum*, *P. vivax*	Not specified	Microscopic method/PCR	Plasma	Commercially available Millipore kit HCYTOMAG-60K-07 (IL-1β, IL-10, IL-6, IL-8, MIP-1α, TNF-α, Luminex technology (Luminex^®^ Corp., Austin, TX, USA))	Significantly higher MIP-1α levels were observed in malaria-infected individuals compared to uninfected individuals.
4	Awandare et al., 2006 [33]	Ghana (2000–2001)	Children (1–10 years old) with acute malaria: Severe malaria without respiratory distress (38), severe malaria with respiratory distress (18), uncomplicated malaria (24)	*P. falciparum*	Severe malaria/uncomplicated malaria	Microscopic method	Plasma	Quantikine ELISA assays (D1000, DTA50, D8050, DMA00, and DMB00; R&D Systems)	MIP-1α: Significantly lower MIP-1α levels were observed in cerebral malaria compared to uncomplicated malaria cases. No difference in MIP-1α between uncomplicated malaria and healthy controls. No difference in MIP-1α between severe malarial anemia and uncomplicated malaria cases. No difference in MIP-1α between cerebral malaria and healthy controls. MIP-1β: No difference in MIP-1β between cerebral malaria, severe malarial anemia, uncomplicated malaria, and healthy controls.
5	Berg et al., 2014 [34]	Mozambique (2011–2012)	Adults (>18 years): Patients with malaria (131), healthy controls (56)	*P. falciparum* (131)*,* mixed *P. falciparum/P. vivax* infections (1)*,* mixed *P. falciparum/P. malariae* infections	Severe malaria/uncomplicated malaria	Microscopic method/RDT/PCR	Plasma	Multiplex cytokine assay (Bio-Plex Human cytokine 27-plex panel; Bio-Rad Laboratories Inc., Hercules, CA, USA)	MIP-1α: No difference in MIP-1α between malaria patients and healthy controls. No difference in MIP-1α between severe malaria and uncomplicated malaria patients. MIP-1β: Significantly higher MIP-1β levels were observed in malaria patients compared to healthy controls. Significantly higher MIP-1β levels were observed in severe malaria compared to uncomplicated malaria patients.
6	Burgmann et al., 1995 [35]	Thailand	Adults (15–65 years): Patients with severe malaria (20), healthy controls (15)	*P. falciparum*	Severe malaria	Microscopic method	Serum	ELISA (Quantikine, R&D Systems)	MIP-1α at admission was higher than healthy controls (the significant difference between groups is unclear).
7	Chaisavaneeyakorn et al., 2003 [36]	Kenya	Pregnant women (98)	*P. falciparum*	Uncomplicated malaria	Microscopic method	Plasma	ELISA (R&D Systems Inc. (Minneapolis, MI, USA.)	MIP-1α: No significant difference in MIP-1α between HIV-negative PM-negative and HIV-negative PM-positive. MIP-1β: No significant difference in MIP-1β between HIV-negative PM-negative and HIV-negative PM-positive.
8	Cox-Singh et al., 2011 [37]	Malaysia (2006–2009)	Adults: *P. knowlesi* (94), *P. vivax* (20), *P. falciparum* (22)	*P. knowlesi*, *P. vivax*, *P. falciparum*	Severe malaria/uncomplicated malaria	Microscopic method/PCR	Serum (plasma in 4 patients)	FluorokineH MAP MultiAnalyte profiling ELISA for LUMINEX Technology (R&D Systems, Inc.) with Bio-Rad Bio-Plex System (Bio-Rad Laboratories Inc.)	MIP-1β: Patients with *P. knowlesi* malaria had significantly lower MIP-1β levels than those infected with *P. falciparum*. No difference in MIP-1β between *P. knowlesi* and *P. vivax* malaria. No significant difference between the *P. vivax* and *P. falciparum* groups.
9	Dieye et al., 2016 [38]	Sénégal (2012–2014)	Adults: Non-cerebral malaria patients (17), cerebral malaria patients (27): (18 survivors, 9 died), control individuals (18)	*P. falciparum*	Severe malaria/uncomplicated malaria	Microscopic method	Serum	Milliplex MAP kit for human cytokine/chemokine magnetic bead panel (catalog # HCYTMAG-60K-PX29; EMD Millipore Corporation, Billerica, MA, USA)	MIP-1α and MIP-1β levels were significantly higher in malaria patients (non-cerebral and/or cerebral malaria) compared to control individuals. MIP-1α and MIP-1β levels were significantly lower in the survivors compared to cerebral malaria patients who died.
10	Dobaño et al., 2020 [39]	Brazil, Colombia, Guatemala, India, Papua New Guinea (2008–2012)	Pregnant women: *P. vivax*-infected (54) and uninfected pregnant women (247)	*P. vivax*	Not specified	Microscopic method/PCR	Plasma	Cytokine Magnetic 30-Plex Panel (Invitrogen, Madrid, Spain)	MIP-1β levels were significantly higher in *P. vivax*–infected pregnant women compared to uninfected women (at recruitment).
11	Frimpong et al., 2022 [46]	Ghana	Children (76): Clinical malaria with no sepsis (33), non-malaria febrile control (20), non-malaria sepsis (23)	*P. falciparum*	Symptomatic malaria	Microscopic method/RDT	Plasma	Human cytokine magnetic 25-plex panel (Thermo Fisher Scientific Corporation, Waltham, MA, USA)	MIP-1α and MIP-1β levels were higher in malaria compared to febrile control (the authors did not show the significant difference).
12	Herbert et al., 2015 [40]	India (2008–2010)	Participants aged 13–72 years: Cerebral malaria (42), cerebral malaria patients with multiple organ dysfunction (41), severe non cerebral malaria (53), multiple organ dysfunction (9), uncomplicated malaria (37), severe sepsis patients (10), viral encephalitis patients (9), healthy subjects (21)	*P. falciparum*	Severe malaria/uncomplicated malaria	Microscopic method/RDT/PCR	Plasma	Luminex multianalytic profiling (MILLIPLEX® MAP human cytokine/chemokine—Premixed 26 Plex, Millipore, USA)	MIP-1α: Significantly higher MIP-1α levels were observed in malaria patients compared to endemic controls. No difference in MIP-1α between severe non-cerebral malaria/multiorgan dysfunction and mild malaria. No difference in MIP-1α between cerebral malaria/cerebral malaria with multiorgan dysfunction and mild malaria. No difference in MIP-1α between severe non-cerebral malaria and multiorgan dysfunction. MIP-1β: No difference in MIP-1β between malaria patients compared to endemic controls. No difference in MIP-1β between severe non-cerebral malaria/multiorgan dysfunction and mild malaria. No difference in MIP-1β between cerebral malaria/cerebral malaria with multiorgan dysfunction and mild malaria. No difference in MIP-1β between severe non-cerebral malaria and multiorgan dysfunction.
13	Jain et al., 2008 [19]	India (2004–2006)	Participants aged < 18 and ≥ 18 years: Cerebral malaria survivors (48), cerebral malaria non-survivors (12), healthy controls (25), mild malaria (48)	*P. falciparum*	Severe malaria/uncomplicated malaria	Microscopic method	Plasma	A multiplex bead-based cytokine immunoassay (MMA) coupled with the Luminex™ system (Austin, TX, USA) and human-specific bead sets (BioRad, San Diego, CA, USA)	MIP-1α: No difference in MIP-1α between cerebral malaria (survivors or non-survivors), mild malaria, and healthy controls. MIP-1β: Significantly higher MIP-1β levels were observed in mild malaria than healthy controls. Significantly higher MIP-1β levels were observed in cerebral malaria survivors compared to healthy controls. There was no difference in MIP-1β between cerebral malaria non-survivors compared to healthy controls. There was no difference in MIP-1β between cerebral malaria (survivors or non-survivors) and mild malaria. There was no difference in MIP-1β between cerebral malaria survivors and non-survivors.
14	John et al., 2006 [18]	Uganda	Children aged 4–12 years: Children with cerebral malaria (88), children with uncomplicated malaria (76), community controls (100)	*P. falciparum*	Severe malaria/uncomplicated malaria	Microscopic method	Serum	Colorimetric bead assay using the Luminex system and human-specific bead sets (R&D Systems).	MIP-1α: Significantly higher MIP-1α in cerebral malaria than in community controls. There was no difference in MIP-1α between cerebral malaria and uncomplicated malaria. MIP-1β: A significantly higher MIP-1β in cerebral malaria than in community controls. There was no difference in MIP-1β between cerebral malaria and uncomplicated malaria.
15	Obeng-Aboagye et al., 2023 [41]	Ghana	Children (57); severe malaria (27), uncomplicated malaria (10), non-malaria-related fever (20)	*P. falciparum*	Severe malaria/uncomplicated malaria	Microscopic method	Plasma	A human cytokine magnetic 25-plex panel (Thermo Fisher Scientific Corporation, Waltham, MA, USA)	Significantly higher MIP-1α/MIP-1β levels were observed in severe malaria compared to febrile controls. There was no difference in MIP-1α/MIP-1β between uncomplicated malaria and febrile controls. There was no difference in MIP-1α/MIP-1β between severe and uncomplicated malaria.
16	Ochiel et al., 2005 [17]	Gabon	Children aged 2–7 years: Severe malaria cases (10), mild malaria cases (10), healthy malaria-exposed subjects (23)	*P. falciparum*	Severe malaria/uncomplicated malaria	Microscopic method	Plasma	Quantitative enzyme-linked immunosorbent assay (Biosource International, Camarillo, CA, USA)	MIP-1α/MIP-1β levels were significantly higher in mild malaria/severe malaria compared to healthy controls. MIP-1α/MIP-1β levels were higher in severe malaria than in mild malaria (but not significantly different between groups).
17	Ong’echa et al., 2011 [42]	Kenya	Children aged 3–30 months: Uncomplicated malaria (31), non-SMA (37), SMA (80)	*P. falciparum*	Severe malaria/uncomplicated malaria	Microscopic method	Plasma	Human cytokine 25-plex antibody bead kit (BioSource International)	There was no difference in MIP-1α/MIP-1β between severe malarial anemia, non-severe malarial anemia, and uncomplicated malaria
18	Royo et al., 2023 [43]	Benin (2018)	Children aged 2–6 years: Cerebral malaria (70): survivors (50), died (20)	*P. falciparum*	Severe malaria	Microscopic method/PCR	Plasma	The Human Premixed Multi-Analyte Kit (LXSAHM-17, R&D Systems, Lille, France)	There was no difference in MIP-1α/MIP-1β (CCL3/CCL4) between cerebral malaria patients who survived and those who died.
19	Suguitan et al., 2003 [44]	Cameroon (1996–2000)	Pregnant women: Malaria positive (89), malaria negative (83)	*P. falciparum*	Not specified	Microscopic method	Plasma	ELISA (DuoSet ELISA Development System; R&D Systems)	There was no difference in MIP-1α/MIP-1β levels between infected and uninfected individuals.
20	Thuma et al., 2011 [45]	Zambia (2001–2005)	Children aged <6 years: Severe malarialanemia (72), cerebral malaria (28), uncomplicated malaria (66)	*P. falciparum*	Severe malaria/uncomplicated malaria	Microscopic method	Plasma	bead-based assay (Human Cytokine/Chemokine Multiplex Immunoassay kits (LINCO Research))	There was no difference in MIP-1α levels between severe malarialanemia, cerebral malaria, and uncomplicated malaria patients.

## Data Availability

All data relating to the present study are available in this manuscript and Tables S1–S4 files.

## Supplementary Materials

The following supporting information can be downloaded at: https://www.mdpi.com/article/10.3390/medicina61040676/s1, Table S1: Search terms; Table S2: Details of included studies; Table S3: Methodological quality of the include studies; Table S4: Subgroup analysis of MIP-1α/MIP-1β between groups of participants.
