# Supplementary material for: A Systematic Review and Meta-Analysis of MIP-1α and MIP-1β Chemokines in Malaria in Relation to Disease Severity
Source: Medicina (Kaunas). 2025 Apr 6;61(4):676. doi: 10.3390/medicina61040676 (PMC12028554; doi:10.3390/medicina61040676)
Supplement: Supplementary file 1 [file medicina-61-00676-s001.zip › medicina-3525769-supplementary/Table S1. Search terms.pdf]

Table S1. Search terms

General keywords

(“Macrophage Inflammatory Proteins” OR “Macrophage Inflammatory Protein” OR “Inflammatory Proteins, Macrophage” OR “Macrophage Inflammatory Protein” OR “Inflammatory Protein, Macrophage” OR “Protein, Macrophage Inflammatory” OR “Macrophage Inflammatory Protein-1” OR “Inflammatory Protein-1, Macrophage” OR “Macrophage Inflammatory Protein 1” OR “Protein-1, Macrophage Inflammatory” OR MIP1 OR “MIP-1” OR “MIP-1α” OR CCL3 OR “chemokine (C-C motif) ligand 3 protein” OR “SCYA3 protein” OR “MIP-1-alpha protein” OR “aminooxypentane-MIP-1alphaP protein” OR “MIP-1-alpha protein” OR “MIP-1-alpha” OR CCL4 OR “C-C motif chemokine ligand 4” OR “chemokine (C-C motif) ligand 4 protein” OR “lymphocyte activation gene-1 protein” OR “Act-2 cytokine” OR “SCYA4 protein” OR “small inducible cytokine A4 protein” OR “MIP-1-beta protein” OR “MIP-1-beta”) AND (malaria OR plasmodium OR “Plasmodium Infection“ OR “Remittent Fever“ OR “Marsh Fever“ OR Paludism)

PubMed 29 September 2024

No.	Key concept	Search terms	Results
1.	Macrophage Inflammatory Protein 1	“Macrophage Inflammatory Proteins”[MeSH Terms] OR “Macrophage Inflammatory Protein”[MeSH Terms] OR “Inflammatory Proteins, Macrophage”[MeSH Terms] OR “Macrophage Inflammatory Protein”[MeSH Terms] OR “Inflammatory Protein, Macrophage”[MeSH Terms] OR “Protein, Macrophage Inflammatory”[MeSH Terms] OR “Macrophage Inflammatory Protein-1”[MeSH Terms] OR “Inflammatory Protein-1, Macrophage”[MeSH Terms] OR “Macrophage Inflammatory Protein 1”[MeSH Terms] OR “Protein-1, Macrophage Inflammatory”[MeSH Terms] OR MIP1[MeSH Terms] OR MIP-1[MeSH Terms] OR MIP-1α[MeSH Terms] OR CCL3[MeSH Terms] OR “chemokine (C-C motif) ligand 3 protein”[MeSH Terms] OR “SCYA3 protein” OR “MIP-1-alpha protein”[MeSH Terms] OR “aminooxypentane-MIP-1alphaP protein” [MeSH Terms] OR “MIP-1-alpha protein”[MeSH Terms] OR “MIP-1-alpha”[MeSH Terms] OR CCL4[MeSH Terms] OR “C-C motif chemokine ligand 4”[MeSH Terms] OR “chemokine (C-C motif) ligand 4 protein”[MeSH Terms] OR “lymphocyte activation gene-1 protein”[MeSH Terms] OR “Act-2 cytokine”[MeSH Terms] OR “SCYA4 protein”[MeSH Terms] OR “small inducible cytokine A4 protein”[MeSH Terms] OR “MIP-1-beta protein”[MeSH Terms] OR “MIP-1-beta”[MeSH	27,373

		Terms] OR “Macrophage Inflammatory Proteins”[All Fields] OR “Macrophage Inflammatory Protein”[All Fields] OR “Inflammatory Proteins, Macrophage”[All Fields] OR “Macrophage Inflammatory Protein”[All Fields] OR “Inflammatory Protein, Macrophage”[All Fields] OR “Protein, Macrophage Inflammatory”[All Fields] OR “Macrophage Inflammatory Protein-1”[All Fields] OR “Inflammatory Protein-1, Macrophage”[All Fields] OR “Macrophage Inflammatory Protein 1”[All Fields] OR “Protein-1, Macrophage Inflammatory”[All Fields] OR MIP1[All Fields] OR “MIP-1”[All Fields] OR “MIP-1α”[All Fields] OR CCL3[All Fields] OR “chemokine (C-C motif) ligand 3 protein”[All Fields] OR “SCYA3 protein”[All Fields] OR “MIP-1-alpha protein”[All Fields] OR “aminooxypentane-MIP-1alphaP protein”[All Fields] OR “MIP-1-alpha protein”[All Fields] OR “MIP-1-alpha”[All Fields] OR CCL4 OR “C-C motif chemokine ligand 4”[All Fields] OR “chemokine (C-C motif) ligand 4 protein”[All Fields] OR “lymphocyte activation gene-1 protein”[All Fields] OR “Act-2 cytokine”[All Fields] OR “SCYA4 protein” OR “small inducible cytokine A4 protein”[All Fields] OR “MIP-1-beta protein”[All Fields] OR “MIP-1-beta”[All Fields]	
2.	Malaria	malaria[MeSH Terms] OR malaria[All Fields] OR malarias[All Fields] OR plasmodium[MeSH Terms] OR plasmodium[All Fields] OR plasmodiums[All Fields] OR "Plasmodium Infection"[All Fields] OR "Remittent Fever"[All Fields] OR "Marsh Fever"[All Fields] OR paludism[All Fields]	128,588
3.	1 AND 2	#1 AND #2	102

Embase 29 September 2024

No.	Key concept	Search terms	Results
1.	Macrophage Inflammatory Protein 1	'macrophage inflammatory proteins'/exp OR 'inflammatory proteins, macrophage' OR 'macrophage inflammatory protein'/exp OR 'inflammatory protein, macrophage' OR 'protein, macrophage inflammatory' OR 'macrophage inflammatory protein-1'/exp OR 'inflammatory protein-1, macrophage' OR 'macrophage inflammatory protein 1'/exp OR 'protein-1, macrophage inflammatory' OR mip1 OR 'mip 1' OR 'mip 1α' OR ccl3 OR 'chemokine (c-c motif) ligand 3 protein' OR 'scya3 protein'/exp OR	65,988

		'aminooxypentane-mip-1alphap protein' OR 'mip-1-alpha protein' OR 'mip-1-alpha' OR 'ccl4'/exp OR 'c-c motif chemokine ligand 4' OR 'chemokine (c-c motif) ligand 4 protein' OR 'lymphocyte activation gene-1 protein' OR 'act-2 cytokine' OR 'scya4 protein'/exp OR 'small inducible cytokine a4 protein' OR 'mip-1-beta protein' OR 'mip-1-beta' OR 'macrophage inflammatory proteins':ti,ab,kw,de OR 'inflammatory proteins, macrophage':ti,ab,kw,de OR 'macrophage inflammatory protein':ti,ab,kw,de OR 'inflammatory protein, macrophage':ti,ab,kw,de OR 'protein, macrophage inflammatory':ti,ab,kw,de OR 'macrophage inflammatory protein-1':ti,ab,kw,de OR 'inflammatory protein-1, macrophage':ti,ab,kw,de OR 'macrophage inflammatory protein 1':ti,ab,kw,de OR 'protein-1, macrophage inflammatory':ti,ab,kw,de OR mip1:ti,ab,kw,de OR 'mip-1':ti,ab,kw,de OR 'mip-1α':ti,ab,kw,de OR ccl3:ti,ab,kw,de OR 'chemokine (c-c motif) ligand 3 protein, human':ti,ab,kw,de OR 'scya3 protein, human':ti,ab,kw,de OR 'mip-1-alpha protein, human':ti,ab,kw,de OR 'aminooxypentane-mip-1alphap protein, human':ti,ab,kw,de OR 'mip-1-alpha protein':ti,ab,kw,de OR 'mip-1-alpha':ti,ab,kw,de OR ccl4:ti,ab,kw,de OR 'c-c motif chemokine ligand 4':ti,ab,kw,de OR 'chemokine (c-c motif) ligand 4 protein':ti,ab,kw,de OR 'lymphocyte activation gene-1 protein':ti,ab,kw,de OR 'act-2 cytokine':ti,ab,kw,de OR 'scya4 protein':ti,ab,kw,de OR 'small inducible cytokine a4 protein':ti,ab,kw,de OR 'mip-1-beta protein':ti,ab,kw,de OR 'mip-1-beta':ti,ab,kw,de	
2.	Malaria	malaria/exp OR plasmodium/exp OR malaria:ti,ab,kw,de OR malarias:ti,ab,kw,de OR plasmodium:ti,ab,kw,de OR plasmodiums:ti,ab,kw,de OR "Plasmodium Infection":ti,ab,kw,de OR "Remittent Fever":ti,ab,kw,de OR "Marsh Fever":ti,ab,kw,de OR paludism:ti,ab,kw,de	164,221
3.	1 AND 2	1 AND 2	322

Scopus 29 September 2024

No.	Key concept	Search terms	Results
1.	Macrophage Inflammatory Protein 1	TITLE-ABS-KEY ("Macrophage Inflammatory Proteins" OR "Macrophage Inflammatory Protein" OR "Inflammatory Proteins, Macrophage" OR "Macrophage Inflammatory Protein" OR "Inflammatory Protein, Macrophage" OR "Protein, Macrophage	57,647

		Inflammatory" OR "Macrophage Inflammatory Protein-1" OR "Inflammatory Protein-1, Macrophage" OR "Macrophage Inflammatory Protein 1" OR "Protein-1, Macrophage Inflammatory" OR mip1 OR "MIP-1" OR "MIP-1α" OR ccl3 OR "chemokine (C-C motif) ligand 3 protein" OR "SCYA3 protein" OR "MIP-1-alpha protein" OR "aminooxypentane-MIP-1alphaP protein" OR "MIP-1-alpha protein" OR "MIP-1-alpha" OR ccl4 OR "C-C motif chemokine ligand 4" OR "chemokine (C-C motif) ligand 4 protein" OR "lymphocyte activation gene-1 protein" OR "Act-2 cytokine" OR "SCYA4 protein" OR "small inducible cytokine A4 protein" OR "MIP-1-beta protein" OR "MIP-1-beta")	
2.	Malaria	TITLE-ABS-KEY (malaria OR plasmodium OR "plasmodium infection" OR "remittent fever" OR "marsh fever" OR paludism)	165,799
3.	1 AND 2	1 AND 2	277

MEDLINE 29 September 2024

No.	Key concept	Search terms	Results
1.	Macrophage Inflammatory Protein 1AND Malaria	(“Macrophage Inflammatory Proteins” OR “Macrophage Inflammatory Protein” OR “Inflammatory Proteins, Macrophage” OR “Macrophage Inflammatory Protein” OR “Inflammatory Protein, Macrophage” OR “Protein, Macrophage Inflammatory” OR “Macrophage Inflammatory Protein-1” OR “Inflammatory Protein-1, Macrophage” OR “Macrophage Inflammatory Protein 1” OR “Protein-1, Macrophage Inflammatory” OR MIP1 OR “MIP-1” OR “MIP-1α” OR CCL3 OR “chemokine (C-C motif) ligand 3 protein” OR “SCYA3 protein” OR “MIP-1-alpha protein” OR “aminooxypentane-MIP-1alphaP protein” OR “MIP-1-alpha protein” OR “MIP-1-alpha” OR CCL4 OR “C-C motif chemokine ligand 4” OR “chemokine (C-C motif) ligand 4 protein” OR “lymphocyte activation gene-1 protein” OR “Act-2 cytokine” OR “SCYA4 protein” OR “small inducible cytokine A4 protein” OR “MIP-1-beta protein” OR “MIP-1-beta”) AND (malaria OR plasmodium OR “Plasmodium Infection“ OR “Remittent Fever“ OR “Marsh Fever“ OR Paludism)	77

Ovid 29 September 2024

No.	Key concept	Search terms	Results
1.	Macrophage Inflammatory Protein 1AND Malaria	("Macrophage Inflammatory Proteins" OR "Macrophage Inflammatory Protein" OR "Inflammatory Proteins, Macrophage" OR "Macrophage Inflammatory Protein" OR "Inflammatory Protein, Macrophage" OR "Protein, Macrophage Inflammatory" OR "Macrophage Inflammatory Protein-1" OR "Inflammatory Protein-1, Macrophage" OR "Macrophage Inflammatory Protein 1" OR "Protein-1, Macrophage Inflammatory" OR MIP1 OR "MIP-1" OR "MIP-1α" OR CCL3 OR "chemokine (C-C motif) ligand 3 protein" OR "SCYA3 protein" OR "MIP-1-alpha protein" OR "aminooxypentane-MIP-1alphaP protein" OR "MIP-1-alpha protein" OR "MIP-1-alpha" OR CCL4 OR "C-C motif chemokine ligand 4" OR "chemokine (C-C motif) ligand 4 protein" OR "lymphocyte activation gene-1 protein" OR "Act-2 cytokine" OR "SCYA4 protein" OR "small inducible cytokine A4 protein" OR "MIP-1-beta protein" OR "MIP-1-beta") AND (malaria OR plasmodium OR "Plasmodium Infection" OR "Remittent Fever" OR "Marsh Fever" OR Paludism) {Including Limited Related Terms}	225

Nursing & Allied Health Premium 29 September 2024

No.	Key concept	Search terms	Results
1.	Macrophage Inflammatory Protein 1 AND Malaria	(“Macrophage Inflammatory Proteins” OR “Macrophage Inflammatory Protein” OR “Inflammatory Proteins, Macrophage” OR “Macrophage Inflammatory Protein” OR “Inflammatory Protein, Macrophage” OR “Protein, Macrophage Inflammatory” OR “Macrophage Inflammatory Protein-1” OR “Inflammatory Protein-1, Macrophage” OR “Macrophage Inflammatory Protein 1” OR “Protein-1, Macrophage Inflammatory” OR MIP1 OR “MIP-1” OR “MIP-1α” OR CCL3 OR “chemokine (C-C motif) ligand 3 protein” OR “SCYA3 protein” OR “MIP-1-alpha protein” OR “aminooxypentane-MIP-1alphaP protein” OR “MIP-1-alpha protein” OR “MIP-1-alpha” OR CCL4 OR “C-C motif chemokine ligand 4” OR “chemokine (C-C motif) ligand 4 protein” OR “lymphocyte activation gene-1 protein” OR “Act-2 cytokine” OR “SCYA4 protein” OR “small inducible cytokine A4 protein” OR “MIP-1-beta protein” OR “MIP-1-beta”) AND (malaria OR	

		plasmodium OR “Plasmodium Infection“ OR “Remittent Fever“ OR “Marsh Fever“ OR Paludism)	

Google Scholar 29 September 2024

No.	Key concept	Search terms	Results
1.	Macrophage Inflammatory Protein 1 AND Malaria	Macrophage Inflammatory Protein 1 AND malaria	The first 200 articles
