# Supplementary material for: A Systematic Review and Meta-Analysis of MIP-1α and MIP-1β Chemokines in Malaria in Relation to Disease Severity
Source: Medicina (Kaunas). 2025 Apr 6;61(4):676. doi: 10.3390/medicina61040676 (PMC12028554; doi:10.3390/medicina61040676)
Supplement: Supplementary file 1 [file medicina-61-00676-s001.zip › medicina-3525769-supplementary/Table S4. Subgroup analysis.pdf]

Table S4. Subgroup analysis of MIP-1α between groups of participants

Plasmodium-infected versus *Plasmodium*-uninfected individuals				
**Subgroup**	**Test for subgroup differences**	**SMD (95% CI)**	**I^2 (%)**	**Number of studies**
**Study design**	0.0922			
Cross-sectional study		1.9683 [0.4453; 3.4912]	96.8	4
Cohort study		0.5328 [-0.1545; 1.2201]	N/A	1
**Continent**	0.4469			
Africa		1.8214 [0.1733; 3.4694]	97.1	4
South America		1.1298 [0.4513; 1.8083]	N/A	1
**Participants**	< 0.0001			
Pregnant women		1.2585 [0.4111; 2.1059]	88.1	3
Children		0.6125 [0.0873; 1.1376]	N/A	1
Adults		4.1460 [3.6076; 4.6845]	N/A	1
***Plasmodium* species**	0.3208			
P. falciparum		1.0735 [0.1287; 2.0182]	92.9	3
Non-*P. falciparum*		2.6453 [-0.3106; 5.6011]	97.9	2
**Diagnostic method for malaria**	< 0.0001			
Microscopy		1.0735 [0.1287; 2.0182]	92.9	3
Microscopy/PCR		1.1298 [0.4513; 1.8083]	N/A	1
Microscopy/RDT/PCR		4.1460 [3.6076; 4.6845]	N/A	1
**Methods for MIP-1α**	0.3208			
ELISA		1.0735 [0.1287; 2.0182]	92.9	3
Bead-based assay		2.6453 [-0.3106; 5.6011]	97.9	2
**Blood samples for MIP-1α**	N/A			
Plasma		1.6866 [0.3777; 2.9955]	95.0	5
Serum	N/A	N/A	N/A	N/A

Abbreviations: ELISA, enzyme-linked immunosorbent assay; RDT, rapid diagnostic test; CI, confidence interval; SMD, standardized mean difference; PCR, polymerase chain reaction; N/A, not assessed.

Table S4. Subgroup analysis of MIP-1β between groups of participants

Plasmodium-infected versus *Plasmodium*-uninfected individuals				
**Subgroup**	**Test for subgroup differences**	**SMD (95% CI)**	I^2 **(%)**	**Number of studies**
**Study design**	0.0739			
Cross-sectional study		1.7427 [1.0242; 2.4611]	91.1	3
Cohort study		0.8266 [0.1241; 1.5291]	N/A	1
**Continent**	N/A			
Africa		1.5413 [0.8744; 2.2082]	88.8	4
South America		N/A	N/A	N/A
**Participants**	< 0.0001			
Pregnant women		1.1602 [0.8796; 1.4408]	4.3	2
Children		1.6007 [1.0248; 2.1765]	N/A	1
Adults		2.4233 [2.0143; 2.8323]	N/A	1
***Plasmodium* species**	< 0.0001			
P. falciparum		1.2412 [1.0021; 1.4804]	29.9	3
Non-*P. falciparum*		2.4233 [2.0143; 2.8323]	N/A	1
**Diagnostic method for malaria**	< 0.0001			
Microscopy		1.2412 [1.0021; 1.4804]	29.9	3
Microscopy/RDT/PCR		2.4233 [2.0143; 2.8323]	N/A	1
**Methods for MIP-1β**	< 0.0001			
ELISA		1.2412 [1.0021; 1.4804]	29.9	3
Bead-based assay		2.4233 [2.0143; 2.8323]	N/A	1
**Blood samples for MIP-1β**	N/A			
Plasma		1.5413 [0.8744; 2.2082]	88.8	4
Serum		N/A	N/A	N/A
